# Exploring the Complex Landscape of Spine Brucellosis

**DOI:** 10.7759/cureus.51761

**Published:** 2024-01-06

**Authors:** Hussam Abu Nowar, Alaa Al Dalahmeh, Maha Alrabadi, Saif Jabali, Martin Kakich, Najib Alqsous, Omar Adaileh, Soha Kannan, Anees Hjazeen, Rami Alqroom

**Affiliations:** 1 Neurosurgery, King Hussein Medical Center - Royal Medical Services, Amman, JOR; 2 Radiology, King Hussein Medical Center - Royal Medical Services, Amman, JOR; 3 Community Medicine, Queen Rania Medical Center - Royal Medical Services, Amman, JOR; 4 Psychiatry, King Hussein Medical Center - Royal Medical Services, Amman, JOR; 5 Directory of Royal Medical Services, King Hussein Medical Center, Amman, JOR; 6 Community Health Nursing, Jordanian Royal Medical Services, Amman, JOR

**Keywords:** intracellular coccobacilli, epidural abscess, spondylitis, gram-negative, spine brucellosis

## Abstract

Introduction: Brucellosis is an infectious illness contracted by humans through the consumption of unpasteurized milk and dairy items. *Brucella *organisms are diminutive, gram-negative, non-motile, and non-spore-forming facultative intracellular, aerobic intracellular coccobacilli residing within the reproductive organs of host animals, leading to conditions such as abortions and sterility. Numerous species of *Brucella* have been identified, with the following four exhibiting varying degrees of pathogenicity in humans: *Brucella melitensis* (originating from sheep), *Brucella suis* (associated with pigs), *Brucella abortus* (linked to cattle), and *Brucella canis* (found in dogs). *B. melitensis* and *B. suis* demonstrate the highest level of pathogenicity, while *B. abortus* and *B. canis* exhibit a more moderate degree of pathogenicity. The illness can progress to systemic involvement, with the musculoskeletal system being particularly susceptible to complications. Spinal epidural abscess is an infrequent complication that may occur during spondylitis caused by *Brucella *species. Epidural abscesses most commonly affect the lumbar vertebrae, while their involvement in the cervical spine is relatively rare.

Objective: In this review, we explore spine brucellosis, covering its epidemiology, pathogenesis, clinical manifestations, diagnostics, and evolving treatments. We aim to enhance early detection, timely intervention, and patient outcomes.

Patients and methods: This retrospective chart analysis was conducted by revising all medical files for patients in whom spinal brucellosis was diagnosed and managed.

Results: This study was conducted at King Hussein Medical Center (KHMC), Jordan, and included a total of 20 patients who were diagnosed with spine brucellosis during the study period. Within the final cohort, 65% of the patients were male (13/20), with an average age at diagnosis of 47.53±14.98 years (ranging from 4 to 61 years). The female group, on the other hand, had an average age at diagnosis of 51.12±15.55 years (ranging from 3 to 58 years). Statistical analysis of the data revealed no significant demographic differences between the two groups. Regarding the co-morbidities, no statistical differences were observed between the two groups. Examining the occupational status of the two groups, no differences were observed. Also, no differences were observed between the two groups regarding the residence place, or the spinal segment involved.

Conclusion: Spine brucellosis, although uncommon, poses a complex clinical challenge. Early diagnosis and a multidisciplinary approach are crucial for effective management. Further research is needed to refine diagnostic tools and treatment guidelines for spine brucellosis.

## Introduction

Brucellosis is an infectious ailment brought about by various *Brucella *species, which goes by several aliases such as remitting fever, undulant fever, Mediterranean fever, Maltese fever, Gibraltar fever, Crimean fever, goat fever, and Bang disease. This illness is contracted by humans through the consumption of unpasteurized milk and dairy items, ingestion of inadequately cooked meat, or via skin contact with livestock [1-3]. Brucella organisms are diminutive, gram-negative, non-motile, and non-spore-forming facultative intracellular, aerobic intracellular coccobacilli residing within the reproductive organs of host animals, leading to conditions such as abortions and sterility. They are excreted in various bodily fluids of these animals, including urine, milk, and placental fluid. Numerous species of *Brucella *have been identified, with the following four exhibiting varying degrees of pathogenicity in humans: *Brucella melitensis* (originating from sheep), *Brucella suis* (associated with pigs), *Brucella abortus* (linked to cattle), and *Brucella canis* (found in dogs). *B. melitensis* and *B. suis* demonstrate the highest level of pathogenicity, while *B. abortus *and *B. canis *exhibit a more moderate degree of pathogenicity [2,4,5]. More rarely, it can spread through the air or direct contact with infected animals [6]. The illness can progress to systemic involvement, with the musculoskeletal system particularly susceptible to complications. These musculoskeletal complications encompass arthritis, bursitis, sacroiliitis, spondylitis, and osteomyelitis. It is worth noting that a spinal epidural abscess is an infrequent complication that may occur during spondylitis caused by *Brucella *species [7]. Spine brucellosis is a relatively rare but potentially debilitating form of the disease [8]. Epidural abscesses most commonly affect the lumbar vertebrae, while their involvement in the cervical spine is relatively rare. The management of spinal epidural abscesses is a subject of debate. Some cases have been successfully treated with antibiotics alone, particularly in patients with stable neurological conditions. However, when there are signs of spinal cord compression, it constitutes a neurosurgical emergency due to the potential for rapid and progressive paralysis [9,10].

In this study, we explore spine brucellosis, covering its epidemiology, pathogenesis, clinical manifestations, diagnostics, and evolving treatments. We aim to enhance early detection, timely intervention, and patient outcomes. We provide insights into the latest developments and future research directions in this complex condition.

## Materials and methods

Ethics

Patient reports from the electronic hospital database over an eight-year period (2015-2023) were analyzed. This study was registered by the Institutional Ethics Committee, Royal Medical Services (IRB: 7/10/2023) on the 3rd of October 2023. As this study was a retrospective analysis, the requirement for patient consent was waived. All the participants’ data handling was accomplished according to the Declaration of Helsinki (2013) and the Health Insurance Portability and Accountability (HIPAA) Acts.

Patients

Using the patient management protocols from the hospital’s database, we conducted a retrospective analysis of the patients admitted and managed as a case of spinal brucellosis during the designated study period. In this research, we collected data from the electronic database documentation, which included the following details: patient demographics (including ID number, name, age, and gender), the clinical presentation, the diagnostic drill, the type of treatment, spine segment involved, occupation, and residency region.

Study design

This retrospective chart analysis was conducted by revising all consecutive patients and their medical files for whom spinal brucellosis was suspected, diagnosed, and managed. The study's inclusion criteria encompass individuals displaying a suspected picture of brucellosis, characterized by a history involving fever, sweating, and/or joint pain. Additionally, those with confirmed *Brucella *isolation or *Brucella *seropositivity ascertained through the Rose Bengal test and confirmed by enzyme-linked immunosorbent assay (ELISA) are included. Residence in Jordan, spinal involvement, and a minimum follow-up period of at least three months are also requisites for inclusion. Conversely, exclusion criteria involve individuals who are travelers from other countries and those lacking either radiological evaluation or clinical data.

Statistical analysis

Data was registered into the Microsoft Excel sheet (Microsoft® Corp., Redmond, WA), and analysis was performed using the statistical software Statistical Package for the Social Sciences (IBM SPSS Statistics for Windows, IBM Corp., Version 28.0, Armonk, NY). The categorical data were expressed in frequency and percentages, while the scale data were stated employing descriptive statistics such as mean and standard deviation for continuous variables. The Chi-square of independence was used to associate categorical data, while the Mann-Whitney test was used to assess mean differences of scale data between two clusters. We employed Freeman-Holton's extension to Fisher's Exact test to model the data due to the exceptionally low sample size, for exploratory analyses to examine the impact of demographic variables and surgical characteristics related to cancellations on patients' perceptions of the healthcare system and their quality of life. We considered a statistically significant result when the Alpha level, set at 0.05, was reached.

## Results

This study was conducted at King Hussein Medical Center (KHMC), Jordan, and included a total of 20 patients who were diagnosed with spine brucellosis during the study period.

Within the final cohort, 65% of the patients were male (13/20), with an average age at diagnosis of 47.53±14.98 years (ranging from 4 to 61 years). Females, on the other hand, had an average age at diagnosis of 51.12±15.55 years (ranging from 3 to 58 years) as shown in (Table 1).

**Table 1:** Demographic data of patients

Parameters	Female group (N=7) (35%)	Male group (N=13) (65%)	P value
Age (years)	47.57±15.54	46.54±11.28	0.847
Body mass index (kg/m^2^)	28.6±5.9	29.0±4.7	0.642
Diabetes	1(14.28%)	3(23.07%)	1
Hypertension	1(14.28%)	2(15.38%)	1
Smoker	3(42.86%)	9(69.23%)	0.356
Occupation			0.13
Dairy Factory	1(14.28%)	4(30.77%)	
Shepherd	0(0%)	5(3.46%)	
Veterinarian	1(14.28%)	2(15.38%)	
Farmer	1(14.28%)	1(7.69%)	
Preschool and School Student	1(14.28%)	1(7.69%)	
Housewife and Retired	2(28.57%)	0(0%)	
Other Occupations	1(14.2%)	0(0%)	
Place of Residence			1
Urban	1(14.28%)	3(23.07%)	
Rural	6(5.71%)	10(76.92%)	
Spine Involvement			1
Cervical	3(42.86%)	6(46.15%)	
Lumbar	4(57.14%)	7(53.84%)	

Statistical analysis of the data revealed no significant differences were observed between the two groups. Regarding the co-morbidities, no statistical differences were observed between the two groups.

**Figure 1:**
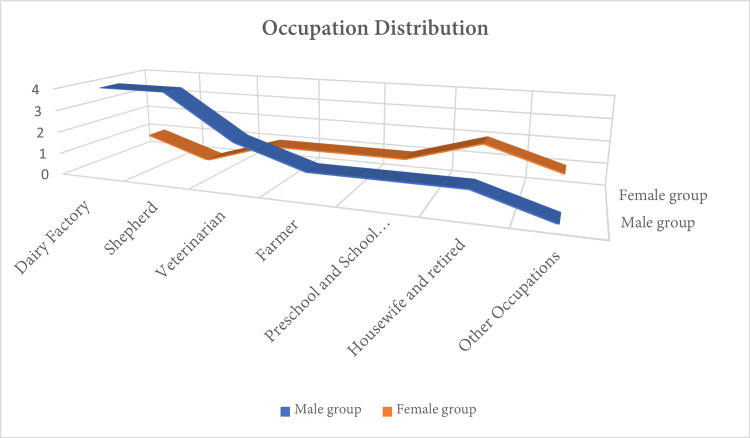
A chart showing the occupation distribution between two groups.

Examining the occupational status of the two groups, no differences were observed (Figure 1).

**Figure 2:**
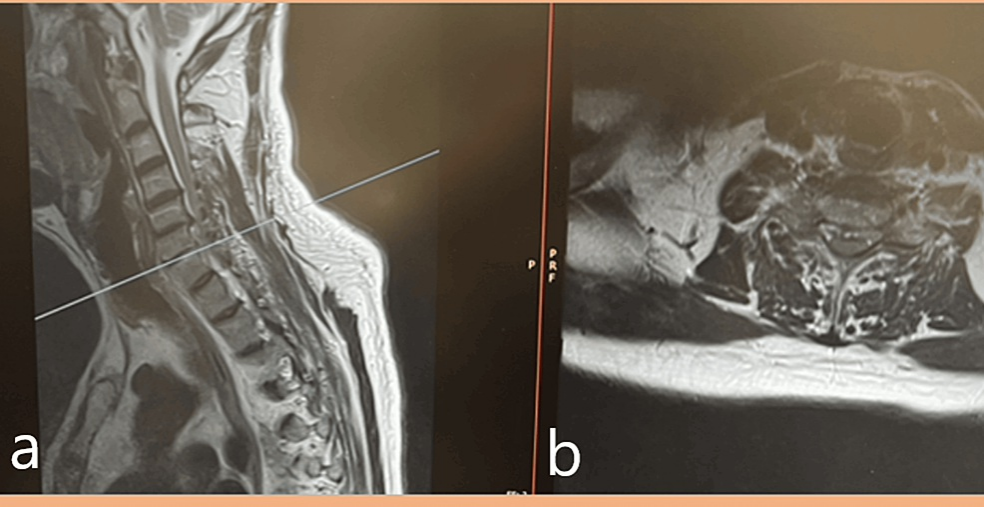
MRI images showing (a) cervical MRI, sagittal view, T2 sequence; and (b) axial view of the same patient. Both showing the epidural collection due to spine involvement of spinal brucellosis.

Also, no differences were observed between the two groups regarding the residence place, or the spinal segment involved. Among the imaging techniques available, MRI is the preferred choice for diagnosing spinal brucellosis due to its high sensitivity in detecting the condition (Figure 2).

The lack of significant differences between the two groups in this study can be attributed to the small sample size employed during data collection.

## Discussion

Human brucellosis, caused by various species of the genus *Brucella*, is a zoonotic infection that predominantly affects livestock and can be transmitted to humans [11-13]. Approximately 500,000 cases of brucellosis are reported annually worldwide, most of which occur in developing countries [14]. In many Middle Eastern countries, including Jordan, human brucellosis is endemic. However, there is a notable absence of up-to-date information regarding the disease's prevalence and overall impact in these regions. Most of the existing studies are predominantly case-control investigations, and there is a limited availability of studies that specifically address brucellosis incidence and seroprevalence [15]. In the study population of Jordan, the overall seroprevalence of *Brucella *infection was found to be 6.7% (with a 95% confidence interval ranging from 5.2% to 8.5%). Notably, a higher seroprevalence rate of 9.3% was observed among immunocompetent children aged 15 years or younger in Jordan [15,16]. Risk factors for brucellosis include immune-compromised conditions like diabetes mellitus, alcoholism, chronic renal failure, cancer, and acquired immunodeficiency syndrome. Additionally, spinal procedures such as epidural anesthesia or analgesia, as well as spinal surgery or trauma, can also increase the risk of infection [17,18]. Additionally, engaging in activities like working with ruminant animals and coming into contact with their manure, as well as milking animals and consuming milk and its products, are strongly linked to brucellosis. Significant risk factors for brucellosis include milking animals and consuming raw cheese, whereas the consumption of cow's milk and boiled feta cheese is associated with a reduced risk of contracting brucellosis (Figure 1) [19].

Human brucellosis can impact any organ or system in the body. *Brucella *can enter the bloodstream through the lymph nodes, gradually disseminating throughout the body [20]. The most common complication involves the musculoskeletal system, leading to conditions like arthritis, spondylitis, and sacroiliitis. These manifestations are accompanied by various non-specific clinical symptoms such as fever, chills, muscle and joint discomfort, headaches, and sweating [21]. It is important to note that some spine-related diseases, including tuberculosis (TB) and pyogenic osteomyelitis, may share radiological similarities with spondylitis in their diagnosis [22].

Among the various clinical presentations, spine brucellosis is a relatively rare but potentially debilitating form of the disease. This infectious disease has been a persistent threat to both animal and human health, leading to significant economic losses in the livestock industry and causing substantial morbidity in affected individuals [23]. Spinal vertebrae are frequently affected by brucellosis in musculoskeletal forms. Studies indicate that 2-54% of *Brucella *infections involve the spine, with the lumbar spine being the most commonly affected [22]. Spine brucellosis incidence varies by geography, often reflecting livestock brucellosis prevalence. Cultural and dietary practices can also impact the incidence. While it is distributed globally, the highest burden of spine brucellosis is observed in regions such as the Mediterranean basin, West Asia, the Middle East, South America, Central America, and sub-Saharan Africa [24]. Spine brucellosis can manifest in several ways, from subclinical forms with minimal symptoms to severe cases with neurological deficits [25].

Accurate and timely diagnosis of spine brucellosis is a critical step in effective management. However, it is often hindered by several challenges. The non-specific clinical symptoms and the rarity of spine brucellosis can lead to misdiagnosis or delayed diagnosis. Another challenging issue arises due to the similarity of its symptoms with those of other chronic conditions, such as TB and pyogenic osteomyelitis. The primary methods used to diagnose spinal brucellosis rely on a combination of factors, including the clinical presentation of the disease, the patient's history of contact with potential infection sources, findings from spinal imaging studies, and laboratory investigations [26]. Among the imaging techniques available, MRI is the preferred choice for diagnosing spinal brucellosis due to its high sensitivity in detecting the condition (Figure 2).

A spinal MRI may reveal signs of spondylitis, spondylodiscitis, or an epidural abscess, often accompanied by a narrowing of the spinal canal [27]. It is important to consider other potential diagnoses when interpreting these findings, such as pyogenic osteomyelitis of the spine, degenerative disc herniation, epidural abscess, and traumatic spondylolisthesis.

Additionally, laboratory tests, including serological assays and microbial cultures, may produce false-negative results. *Brucella *serological assays often exhibit a propensity to produce affirmative outcomes and possess a notable diagnostic efficacy. The benchmark modality endorsed by the World Health Organization (WHO) entails the utilization of the *Brucella *agglutination test. Additionally, the Rose Bengal Test represents a swift, highly responsive, and exceptionally precise diagnostic approach. This methodology is executed employing a Rose Bengal-stained bacterial solution within a buffered acidic milieu [28,29]. As a result, the definitive diagnosis of spine brucellosis often requires a combination of clinical suspicion, imaging studies, serological tests, and sometimes microbiological culture. These diagnostic challenges underscore the need for increased awareness and research to improve diagnostic accuracy [30].

The cornerstone of spine brucellosis treatment is a prolonged course of appropriate antibiotics. The choice of antibiotic regimen, duration of treatment, and the need for surgical intervention must be individualized based on the patient's condition, including the extent of vertebral involvement and the presence of complications [31]. Surgical intervention may be necessary in cases with abscess formation, vertebral destruction, or neurological compromise. The decision to pursue surgical treatment should be made collaboratively between infectious disease specialists and spine surgeons, considering the specific clinical and radiological findings [32]. This multifaceted approach to treatment reflects the complexity of spine brucellosis management and underscores the importance of a multidisciplinary healthcare team.

In summary, spine brucellosis, though infrequent, represents a challenging and potentially devastating manifestation of brucellosis. By comprehensively examining these aspects, healthcare practitioners can gain a deeper understanding of spine brucellosis and improve their ability to recognize, diagnose, and manage this complex condition effectively. Further research and collaboration among medical professionals are essential for advancing our knowledge of spine brucellosis and optimizing patient outcomes.

While this study on spine brucellosis offers valuable insights into demographics, clinical characteristics, and diagnostic challenges, several limitations warrant consideration. Primarily, the study's reliance on a small sample size within a specific time frame, drawn from a single hospital database, raises concerns regarding its representativeness and generalizability to broader populations, leading to decreased statistical power and increased susceptibility to random variations. Consequently, small sample sizes may fail to detect true effects that might exist within a larger and more diverse population. The retrospective nature of the analysis might have introduced selection bias and limited the scope of available data, potentially overlooking crucial variables that could impact the understanding of spine brucellosis. Additionally, the exclusion criteria, while necessary for defining the study cohort, might have restricted the inclusivity of certain individuals who could have contributed diverse perspectives or clinical presentations. Moreover, the study's focus on a particular geographic region might limit the applicability of its findings to other areas with different epidemiological or socio-cultural contexts. These limitations underscore the need for larger, more diverse cohorts, prospective study designs, and multi-center collaborations to mitigate biases, enhance the study's robustness, and foster a more comprehensive understanding of spine brucellosis.

## Conclusions

Human brucellosis, prevalent in regions like Jordan, remains a challenge due to limited up-to-date epidemiological data. This study outlined associated risk factors, diverse clinical presentations, and challenges in diagnosis. The intricate landscape of spine brucellosis, although uncommon, poses a challenging and often debilitating aspect of this infectious disease caused by various *Brucella *species.

The multifaceted approach to managing spine brucellosis, involving tailored antibiotic regimens and, in some cases, surgical intervention, reflects the complexity of the condition. Collaboration between infectious disease experts and spine surgeons emerges as a crucial aspect of decision-making for optimal patient care. Ultimately, this study stresses the significance of continued research, collaboration, and a multidisciplinary approach in advancing our knowledge and improving patient outcomes in this challenging domain. Moreover, it underscored the necessity for heightened awareness among healthcare professionals to prevent misdiagnosis, considering the disease's resemblance to other chronic conditions.

## References

[1] Głowacka P, Żakowska D, Naylor K, Niemcewicz M, Bielawska-Drózd A (2018). Brucella - virulence factors, pathogenesis and treatment. Pol J Microbiol.

[2] Hull NC, Schumaker BA (2018). Comparisons of brucellosis between human and veterinary medicine. Infect Ecol Epidemiol.

[3] Bakri FG, AlQadiri HM, Adwan MH (2018). The highest cited papers in brucellosis: identification using two databases and review of the papers’ major findings. Biomed Res Int.

[4] Brown VR, Bowen RA, Bosco-Lauth AM (2018). Zoonotic pathogens from feral swine that pose a significant threat to public health. Transbound Emerg Dis.

[5] Craighead L, Meyer A, Chengat B (2018). Brucellosis in West and Central Africa: a review of the current situation in a changing landscape of dairy cattle systems. Acta Trop.

[6] Köse Ş, Senger SS, Çavdar G, Yavaş S (2011). Case report on the development of a brucellosis-related epidural abscess. J Infect Dev Ctries.

[7] Arkun R, Mete BD (2011). Musculoskeletal brucellosis. Semin Musculoskelet Radiol.

[8] Zhang H, Xie S, Wang Y (2021). A case report of endocarditis and spondylitis caused by Brucella melitensis biovar 3. BMC Infect Dis.

[9] Solera J, Lozano E, Martínez-Alfaro E, Espinosa A, Castillejos ML, Abad L (1999). Brucellar spondylitis: review of 35 cases and literature survey. Clin Infect Dis.

[10] Pathak A, Singh P, Gehlot P, Dhaneria M (2013). Spinal epidural abscess treated with antibiotics alone. BMJ Case Rep.

[11] Walker LR (1999). Veterinary Microbiology. https://www.scirp.org/(S(lz5mqp453ed%20snp55rrgjct55))/reference/referencespapers.aspx?referenceid=1494589.

[12] (1986). Joint FAO/WHO expert committee on brucellosis. World Health Organ Tech Rep Ser.

[13] Ali Adam A, Sheikh Hassan M, Adam Osman A (2022). Spinal brucellosis causing spondylodiscitis. Ann Med Surg (Lond).

[14] Eini P, Keramat F, Hasanzadehhoseinabadi M (2012). Epidemiologic, clinical and laboratory findings of patients with brucellosis in Hamadan, west of Iran. J Res Health Sci.

[15] Obaidat MM, Malania L, Arner RJ, Roess AA (2022). Seroprevalence and risk factors for Brucella infections in Jordan. Am J Trop Med Hyg.

[16] Al-Majali AM, Shorman M (2009). Childhood brucellosis in Jordan: prevalence and analysis of risk factors. Int J Infect Dis.

[17] Ugarriza LF, Porras LF, Lorenzana LM, Rodríguez-Sánchez JA, García-Yagüe LM, Cabezudo JM (2005). Brucellar spinal epidural abscesses. Analysis of eleven cases. Br J Neurosurg.

[18] Shafizad M, Ehteshami S, Shojaei H, Jalili Khoshnoud R (2022). Cervical spine epidural abscess caused by brucellosis: a case report and literature review. Clin Case Rep.

[19] Abo-Shehada MN, Abu-Halaweh M (2013). Risk factors for human brucellosis in northern Jordan. East Mediterr Health J.

[20] Doganay M, Aygen B (2003). Human brucellosis: an overview. Int J Infect Dis.

[21] Hassan-Kadle AA, Osman AM, Shair MA, Abdi OM, Yusuf AA, Ibrahim AM, Vieira RF (2021). Rift Valley fever and Brucella spp. in ruminants, Somalia. BMC Vet Res.

[22] Malavolta N, Frigato M, Zanardi M, Mule R, Lisi L, Gnudi S, Fini M (2002). Brucella spondylitis with paravertebral abscess due to Brucella melitensis infection: a case report. Drugs Exp Clin Res.

[23] Alp E, Doganay M (2008). Current therapeutic strategy in spinal brucellosis. Int J Infect Dis.

[24] Nas K, Gür A, Kemaloğlu MS (2001). Management of spinal brucellosis and outcome of rehabilitation. Spinal Cord.

[25] Alyousef M, Aldoghaither R (2018). First case of cervical epidural abscess caused by brucellosis in Saudi Arabia: a case report and literature review. IDCases.

[26] Applebaum GD, Mathisen G (1997). Spinal brucellosis in a southern California resident. West J Med.

[27] Roushan MR, Ebrahimpour S, Afshar ZM, Babazadeh A (2019). Cervical spine spondylitis with an epidural abscess in a patient with brucellosis: a case report. J Crit Care Med (Targu Mures).

[28] Yagupsky P (1999). Detection of Brucellae in blood cultures. J Clin Microbiol.

[29] Kim DH, Cho YD (2008). A case of spondylodiscitis with spinal epidural abscess due to Brucella. J Korean Neurosurg Soc.

[30] Zhang Y, Zhang Q, Zhao CS (2019). Cervical brucellar spondylitis causing incomplete limb paralysis. Rev Soc Bras Med Trop.

[31] Akpinar O, Guzel M (2020). Spinal stenosis caused by epidural and paraspinal abscess due to brucella Infection. J Pak Med Assoc.

[32] Chen Y, Yao S, He WQ, Zhao X (2021). The application of surgical treatment in spinal brucellosis. Asian J Surg.

